# Personal

**Published:** 1891-02

**Authors:** 


### Personal.

Dr. William C. Krauss, of Buffalo, has been placed upon the editorial staff of the *Journal of Nervous and Mental Disease*, New York, one of the oldest and best journals devoted to this specialty in America.

Dr. Frank W. Low, of Buffalo, read an interesting paper on Phagocytosis, at the Joint Convention (Dentists), held in Rochester, October 27, 1890, which is published in the *Dental Advertiser*, January, 1891.

